# Canopy Position Has a Stronger Effect than Tree Species Identity on Phyllosphere Bacterial Diversity in a Floodplain Hardwood Forest

**DOI:** 10.1007/s00248-020-01565-y

**Published:** 2020-08-06

**Authors:** Martina Herrmann, Patricia Geesink, Ronny Richter, Kirsten Küsel

**Affiliations:** 1 Institute of Biodiversity, Aquatic Geomicrobiology, Friedrich Schiller University Jena, Dornburger Strasse 159, D-07743 Jena, Germany; 2 German Center for Integrative Biodiversity Research (iDiv) Halle-Jena-Leipzig, Deutscher Platz 5e, 04103 Leipzig, Germany; 3 Systematic Botany and Functional Biodiversity, Institute for Biology, Leipzig University, Johannisallee 21, 04103 Leipzig, Germany; 4 Geoinformatics and Remote Sensing, Institute of Geography, Leipzig University, Johannisallee 19a, 04103 Leipzig, Germany

**Keywords:** Phyllosphere, Canopy crane, *Acer pseudoplatanus*, *Quercus robur*, *Tilia cordata*

## Abstract

The phyllosphere is a challenging microbial habitat in which microorganisms can flourish on organic carbon released by plant leaves but are also exposed to harsh environmental conditions. Here, we assessed the relative importance of canopy position—top, mid, and bottom at a height between 31 and 20 m—and tree species identity for shaping the phyllosphere microbiome in a floodplain hardwood forest. Leaf material was sampled from three tree species—maple (*Acer pseudoplatanus* L.), oak (*Quercus robur* L.), and linden (*Tilia cordata* MILL.)—at the Leipzig canopy crane facility (Germany). Estimated bacterial species richness (Chao1) and bacterial abundances approximated by quantitative PCR of 16S rRNA genes exhibited clear vertical trends with a strong increase from the top to the mid and bottom position of the canopy. Thirty operational taxonomic units (OTUs) formed the core microbiome, which accounted for 77% of all sequence reads. These core OTUs showed contrasting trends in their vertical distribution within the canopy, pointing to different ecological preferences and tolerance to presumably more extreme conditions at the top position of the canopy. Co-occurrence analysis revealed distinct tree species-specific OTU networks, and 55–57% of the OTUs were unique to each tree species. Overall, the phyllosphere microbiome harbored surprisingly high fractions of Actinobacteria of up to 66%. Our results clearly demonstrate strong effects of the position in the canopy on phyllosphere bacterial communities in a floodplain hardwood forest and—in contrast to other temperate or tropical forests—a strong predominance of Actinobacteria.

**Electronic supplementary material:**

The online version of this article (10.1007/s00248-020-01565-y) contains supplementary material, which is available to authorized users.

## Introduction

The phyllosphere is an important microbial habitat which spans about 10^8^ km^2^ on a global scale [1]. It is host to central biogeochemical processes as well as plant-microbe interactions that affect plant community dynamics, and ecosystem functioning and productivity [2, 3]. Microbiota on leaf surfaces contribute to biogeochemical processes such as N_2_ fixation [4, 5], nitrification [6], and transformation of C1 compounds or terpenes and monoterpenes released from the plants [7]. Especially in forest canopies, they may play a central role in the bioremediation of air pollutants [8] and are in exchange with atmospheric and cloud microbiota, suggesting important implications for climate regulation [7]. Beyond their role in biogeochemical processes, phyllosphere microbiota are not only passive inhabitants on surfaces of plants but interact with their host in multiple ways [9], resulting in plant-microbe relationships that range from loose associations to defined symbioses [7]. They produce phytohormones or affect the production of these hormones by the plant [10–12] and they improve host resistance against pathogens [13, 14].

However, the phyllosphere is also a harsh environment where microorganisms are exposed to extreme conditions such as high UV radiation, desiccation, rainfall, antimicrobial substances released by leaves, and strong nutrient limitation [7]. Altogether, these environmental parameters positively select for the bacterial taxa that are able to persist on leaves [15]. As a consequence, phyllosphere bacterial diversity has been shown to be much lower than diversity of the rhizosphere, soil, or marine ecosystems [16–18]. Especially in the canopy of large trees, selective environmental forces that restrict microbial diversity are likely to vary along vertical gradients [19] with the severest stress by abiotic parameters presumably acting at the top of the canopy. However, studies addressing intra-individual variability of phyllosphere microbial communities have so far mostly focused on a single tree species such as *Gingko biloba* or *Magnolia grandiflora* [20, 21] or on rather small trees at a maximum height of 6 m [19]. Ongoing colonization of leaves by microorganisms and their continuous removal, e.g., by rain fall, result in complex community dynamics in the forest canopy phyllosphere [7]. It is unclear if microbial taxa differ in their preference for a particular canopy position and how this translates into the spatial heterogeneity of canopy-associated biogeochemical processes. Despite their high relevance for ecosystem functioning, phyllosphere microbiota in forest canopies have so far received comparatively little attention. Factors such as host species identity, leaf age, location in the canopy, light incidence, and microclimate conditions have been identified as central factors shaping the phyllosphere environment in tropical and North American temperate forests [22–27; 3]. However, more insight is needed if the phyllosphere diversity patterns observed for tropical and North American forests, especially the strong effect of host species identity, also apply to European forests with a different tree species composition.

Here, we hypothesize that (i) forest trees harbor species-specific phyllosphere bacterial communities, and that (ii) microbial communities at the treetop are the most distinct, as they are the most exposed to abiotic stress factors. Taking advantage of the Leipzig canopy crane facility located in central Germany, allowing us to sample leave material from up to 33 m height, we compared phyllosphere microbial communities between three different tree species abundant in the Leipzig floodplain forest—*Acer pseudoplatanus* L., *Quercus robur* L., and *Tilia cordata* MILL.—and across three different height levels within the canopy—top, mid, and bottom. Our results revealed clear vertical trends of increasing bacterial diversity and abundances, and changes in community structure from the top of the canopy to mid and bottom canopy, which were further modulated by plant species identity.

## Methods

### Leipzig Floodplain Hardwood Forest and Canopy Crane Facility

Leaf samples were obtained from three tree species—*Q. robur* L. (oak; Qr), *A. pseudoplatanus* L. (maple; Ap), and *T. cordata* MILL. (linden; Tc)—in the Leipzig floodplain hardwood forest, located near the city of Leipzig in Germany (Supplementary Fig. 1a). Situated in the floodplain of the Elster, Pleiße, and Luppe rivers, the Leipzig floodplain forest is one of the largest floodplain forests in Central Europe [22]. Climatic conditions are characterized by warm summers and an annual mean temperature of 8.4 °C with an annual precipitation of 516 mm [23]. The forest consists of the ash-elm floodplain forest (*Fraxino-Ulmetum*) and is dominated by maple (*A. pseudoplatanus* L.), ash (*Fraxinus excelsior* L.), oak (*Q. robur* L.), and hornbeam (*Carpinus betulus* L.), with smaller contribution of linden (*T. cordata* MILL.) and elm (*Ulmus minor* MILL.) [24]. A crane facility (Leipzig Canopy Crane facility, LCC) for the investigation of forest tree canopies was established in this floodplain forest in 2001, allowing access to about 800 tree individuals in up to 33 m height, covering a total area of 1.65 ha (Supplementary Fig. 1a). The estimated age of the trees sampled in this study ranged from 102 to 370 years for oak, 97 to 146 years for maple, and 205 to 240 years for linden (Supplementary Table 1). Different positions within the tree canopy were accessed by using a gondola attached to the crane. We sampled leaf material from the top, mid, and bottom position of the tree canopy. Depending of the height of individual trees, these positions ranged from 27.0 to 30.7 m, 18.6 to 26.3 m, and 12.9 to 23.2 m, respectively (Supplementary Fig. 1b). Samples were obtained from three individuals per tree species. At each canopy position, we sampled leaves in three spatial replicates, that is, leaves were sampled from different twigs which were between 0.2 and 0.5 m apart on the same height level. This way, a total of 81 samples were collected. For each tree individual, canopy position, and spatial replicate, between five and ten leaves were sampled and pooled for further analysis.

### Sampling of Leaf Material and Detachment of Surface-Associated Microbes

Leaves were sampled by clipping off leaves with ethanol-cleaned scissors, followed by immediate transfer to autoclaved polyphenylene ether (PPE) containers. Leaves were stored at ambient temperature (approx. 15 °C) during transport and were immediately processed upon arrival at the laboratory within 2 h. Leaves were amended with 250 ml suspension buffer (0.15 M NaCl, 0.1% Tween 20; [9]) in the autoclaved containers in which leaves had been sampled, subjected to mild sonication (1 min at 10% intensity, turned and another 1 min at 10% intensity), followed by shaking for 20 min at 100 rpm at room temperature. Subsequently, suspensions were filtered through 0.2-μm polyethersulfone filters (Supor, Pall Corporation), and filters were stored at −80 °C until nucleic acid extractions were performed. The remaining leaf material was dried at 50 °C for 1 week for determination of dry weight.

### Nucleic Acid Extraction, Illumina MiSeq Amplicon Sequencing, and Quantitative PCR

DNA was extracted from the filters using the DNeasy PowerSoil Extraction kit (Qiagen) following the manufacturer’s protocol. Filters were cut into smaller pieces to facilitate cell disruption during the bead-beating step. Amplicon sequencing of bacterial 16S rRNA genes was carried out targeting the V3-V4 region with the primer combination Bakt_0341F/Bakt_0785R [25]. PCR amplification, library preparation, and sequencing on an Illumina MiSeq platform using v3 chemistry was performed at LGC (Berlin) as previously described [26]. Abundances of bacterial 16S rRNA genes were determined by quantitative PCR using Brilliant SYBR Green II Mastermix (Agilent Technologies) on a Mx3000P system (Agilent Technologies) and the primer combination Bakt_0341F [25] and Bakt_0799R, which is the reverse complement version of the primer 799F [27] discriminating against chloroplast-derived 16S rRNA genes. For qPCR, we used the following cycling conditions: 10 min at 95 °C, followed by 45 cycles of 30 s at 95 °C, 30 s at 53 °C, and 40 s at 72 °C, and subsequent melting curve analysis. Due to loss of plant material of some samples before determination of leaf dry weight, abundance data are only available for a subset of all samples (see Supplementary Table 2).

### Sequence Analysis

Sequence analysis was carried out using Mothur v1.39.1 [28] following the Schloss MiSeq SOP [29] as previously described [26] along with the SILVA taxonomy reference database v132 [30]. Chimera search was performed using the uchime algorithm implemented in Mothur. Operational taxonomic units (OTUs) were assigned on a 0.03 distance level using the vsearch algorithm. We obtained 6,037,303 high-quality sequence reads across 81 samples with read numbers per sample ranging from 1610 to 198,790. For further statistical analysis, read numbers were normalized to the same number for all samples (11,188 reads) using the sub.sample function implemented in Mothur, resulting in the exclusion of two samples with too low read numbers from the data set (Ap6 and Qr18). Sample Tc12 was also excluded from further analysis because a large part of the sample had been lost during filtration, making the bacterial community composition less comparable. Sequences obtained in this study have been submitted to the European Nucleotide Archive (ENA) under the study accession number PRJEB36420, sample accession numbers SAMEA6502636–SAMEA6502715.

### Statistical Analysis

Variation of bacterial species richness (number of observed and estimated OTUs (Chao)) across canopy positions and tree species was analyzed using two-factorial ANOVA followed by Tukey’s post hoc test. Vertical changes in relative abundances of phylum-level taxa between top, mid, and bottom canopy position were analyzed by linear regression. Principal coordinate analysis (PCoA) and PERMANOVA analysis based on Bray-Curtis dissimilarities was employed to assess effects of tree species identity and position in the canopy on phyllosphere bacterial community composition. PERMANOVA analysis was run with 999 permutations. For PCoA analysis, only OTUs with at least 10 reads across all samples were included. To further investigate the effect of tree species on the phyllosphere bacterial communities, we merged all the OTUs observed in association with a given tree species in the different samples (tree individuals, canopy positions, and spatial replicates) to one OTU pool and compared the resulting three tree species-dependent OTU pools to each other using Venn diagrams in Mothur. To assess whether the fraction of OTUs shared between top, mid, and bottom canopy position was different, we first pooled all the OTUs found in association with one tree individual and canopy position and determined the shared OTUs between top/mid, top/bottom, and mid/bottom canopy for each tree individual. Values from all nine tree individuals were used to analyze if top/mid, top/bottom or mid/bottom shared fractions were significantly different from each other, which was done using the Mann-Whitney *U* test. Finally, we followed distribution patterns of the 20 most abundant OTUs across tree species and position in the canopy by subjecting relative abundances of these OTUs to hierarchical clustering based on Bray-Curtis dissimilarities, combined with visualization using a heatmap. Except for PERMANOVA analysis, which was done in R [31] using the Adonis functions from the R package vegan [32], all these calculations were carried out using the software PAST v3.11 [33]. This software was also used for the generation of box and whisker plots.

Co-occurrence network analysis was done using the MENA platform [34]. Networks were constructed for the phyllosphere microbiome of each tree species, including samples from all three canopy positions, tree individuals, and spatial replicates per tree species. Only OTUs with more than 20 sequence reads across all samples per tree species were included. Network calculations were based on Spearman rank correlation coefficients without log transformation of the data. Similarity thresholds were 0.68, 0.65, and 0.67 for maple, oak, and linden, respectively. Networks were graphically refined using Cytoscape 3.7.2.

## Results

### Effect of Canopy Position and Tree Species on Phyllosphere Bacterial Communities

**Fig. 1:**
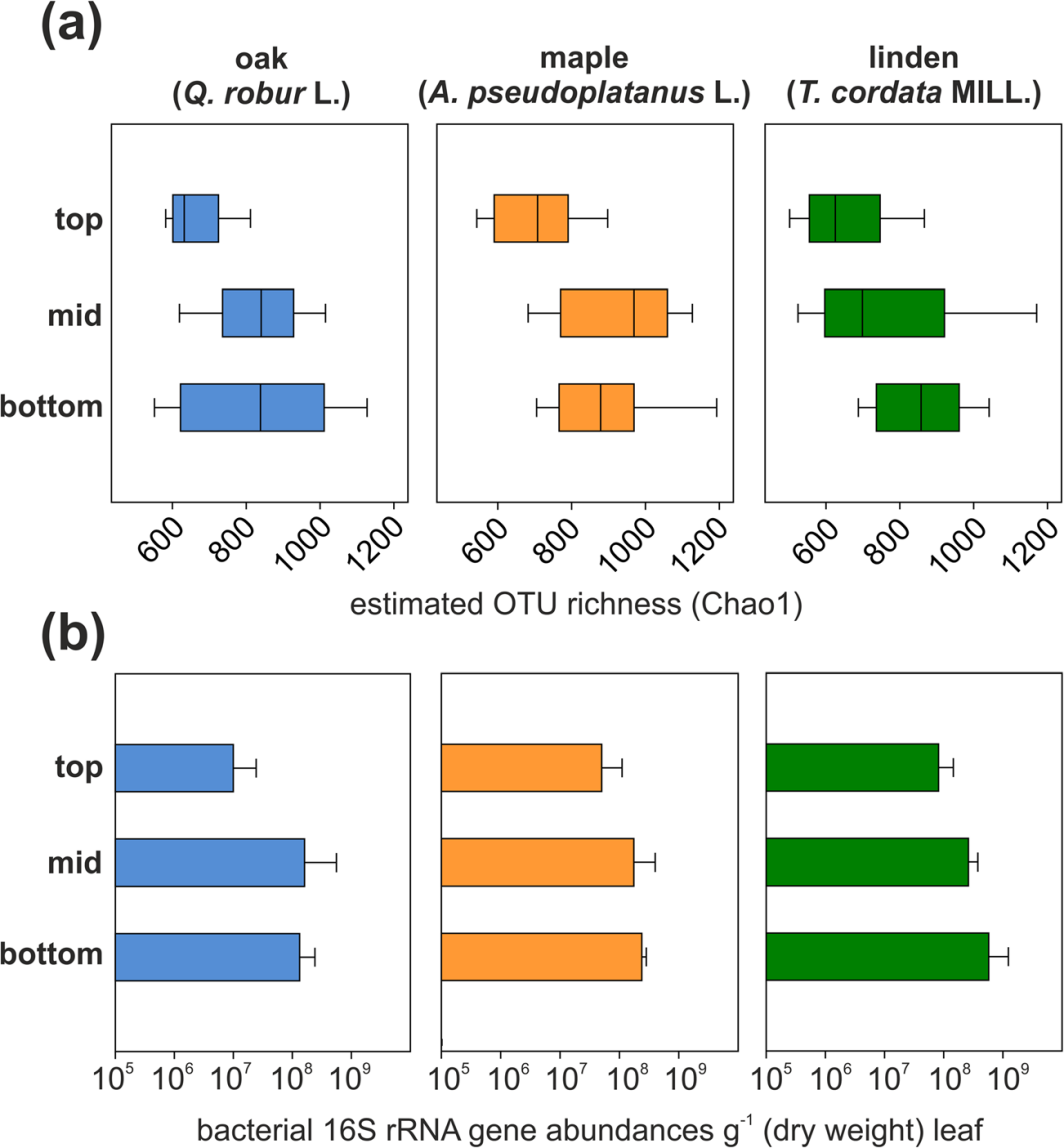
**a** Estimated bacterial species richness and **b** abundance of bacterial 16S rRNA genes per g leaf (dry weight) in the canopy of *Q. robur* L. (left panel), *A. pseudoplatanus* L. (mid panel), and *T. cordata* MILL. (right panel) with positions in the canopy categorized as “top,” “mid,” and “bottom.” Data are means (± standard deviation) of results obtained from three tree individuals with three replicates per sampled canopy area. For gene abundances, a reduced number of samples is available (see Supplementary Table 2)

Amplicon sequencing of bacterial 16S rRNA genes revealed clear vertical trends in OTU richness and community composition from the top to the bottom canopy position, further modulated by the tree species. The number of observed and estimated (Chao estimator) species-level OTUs tended to increase from the top of the canopy towards the mid position (Fig. 1a). For the phyllosphere of linden, the increase in bacterial OTU numbers was already visible between the mid and the bottom position of the canopy while such a trend was less obvious for the other two tree species. Median values of observed (estimated) OTU numbers ranged from 264 to 331 (607 to 655) at the top of the canopy, from 332 to 400 (729 to 937) at the mid position, and from 393 to 429 (875 to 933) at the bottom of the canopy with the highest numbers observed in association with maple. Overall, canopy position had a significant effect on both observed (*F* = 19.66, *p* < 0.001) and estimated OTU numbers (*F* = 11.86, *p* < 0.001), while the effect of tree species was only significant for the observed OTUs (*F* = 3.64, *p* = 0.03). Interactions between these two factors were not significant.

Abundances of bacterial 16S rRNA genes per g dry weight leaf showed a similar trend as bacterial OTU richness, with lower abundances at the top of the canopy compared to the mid or bottom position. Both canopy position (*F* = 8.717, *p* < 0.001) and tree species (*F* = 9.09, *p* < 0.001) had a significant effect on bacterial abundances. However, these data rather represent trends, as for some of the samples, especially from the top of the canopy, only a reduced number of replicates is available. Across all tree species, individuals, and position in the canopy, bacterial 16S rRNA gene abundances ranged from 1.9 × 10^6^ to 1.3 × 10^9^ g^−1^ (dry weight) (Fig. 1b). Abundances observed in association with linden were significantly higher than the numbers in association oak (Tukey’s post hoc test, *p* < 0.001) or maple (*p* < 0.001).

**Fig. 2:**
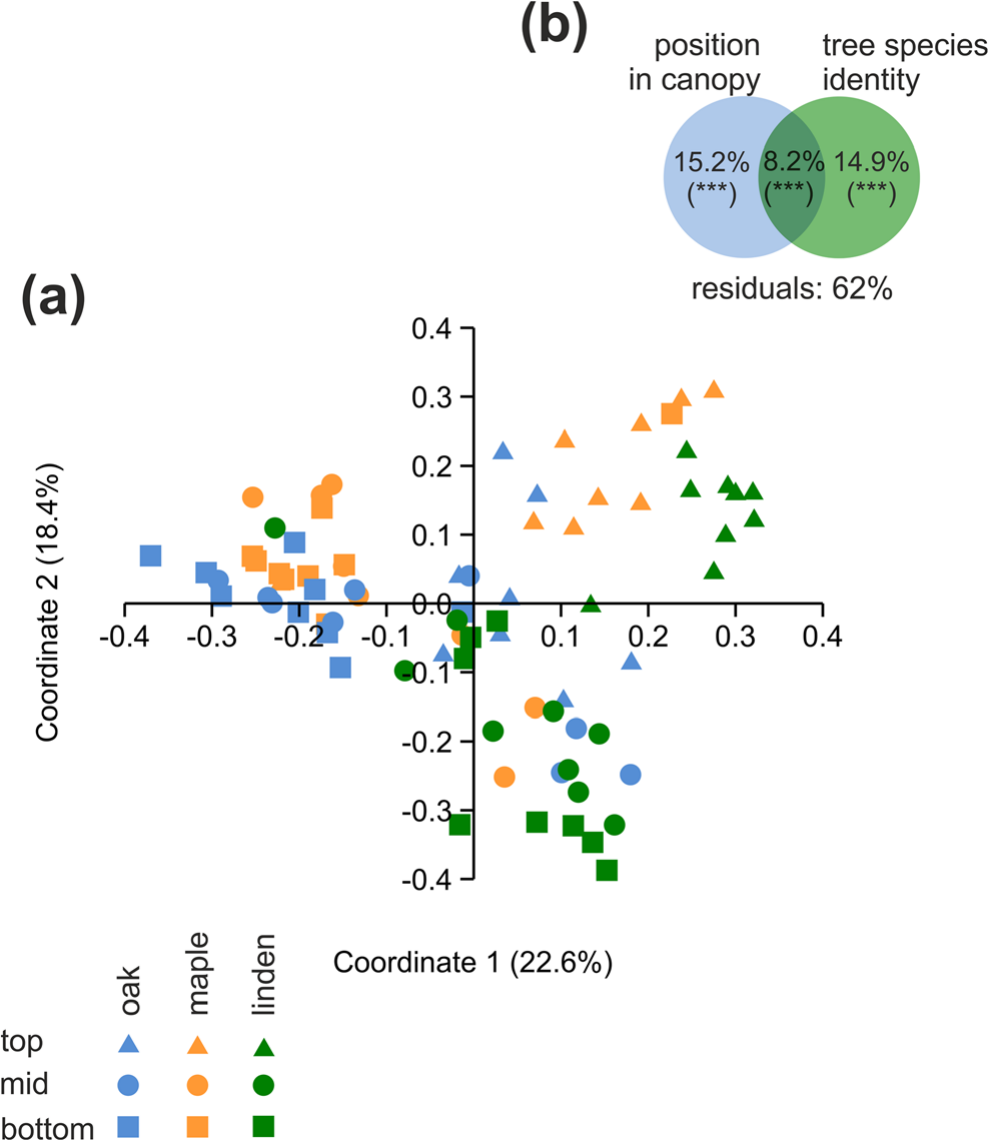
**a** Principal  coordinate analysis of phyllosphere bacterial communities across three tree species and top, mid, and bottom positions of the canopy. **b** Variation partitioning resulting from PERMANOVA analysis. Analyses were based on distribution patterns of species-level OTUs using Bray-Curtis dissimilarities. Colors denote tree species, symbols denote position within the tree canopy

In line with these findings, PERMANOVA analysis based on Bray-Curtis dissimilarities revealed that position in the canopy as well as tree species had a significant effect on phyllosphere bacterial community structure (*p* < 0.001) with canopy position explaining 15.2% and tree species identity explaining 14.9% of the total community variation (Fig. 2b; Supplementary Table 3). In addition, interactions between these two factors also had a significant effect (*p* < 0.001). The observed height- and tree species-dependent trends in bacterial OTU richness were reflected by clustering patterns of samples using principal coordinate analysis. Here, especially for linden and maple, samples obtained from the top of the canopy clustered separate from samples obtained from mid or bottom positions with clear differences in OTU composition between these two tree species (Fig. 2a). In contrast, for the mid and bottom positions of the canopy, we observed minor clustering of communities according to tree species but also large overlaps. For each tree species, communities of the mid and bottom positions were more similar to each other than they were to the communities at the top of the canopy. In fact, for all three tree species together, the fraction of OTUs shared between the top and mid canopy positions (15–19.4%) or the top and bottom canopy positions (15.5–20.5%) was significantly lower than the fraction of OTUs shared between the mid and bottom positions (19.4–25.1%; Mann-Whitney *U* test, *p* = 0.00172) (Supplementary Fig. 2).

### Composition of the Hardwood Forest Canopy Microbiome

**Fig. 3:**
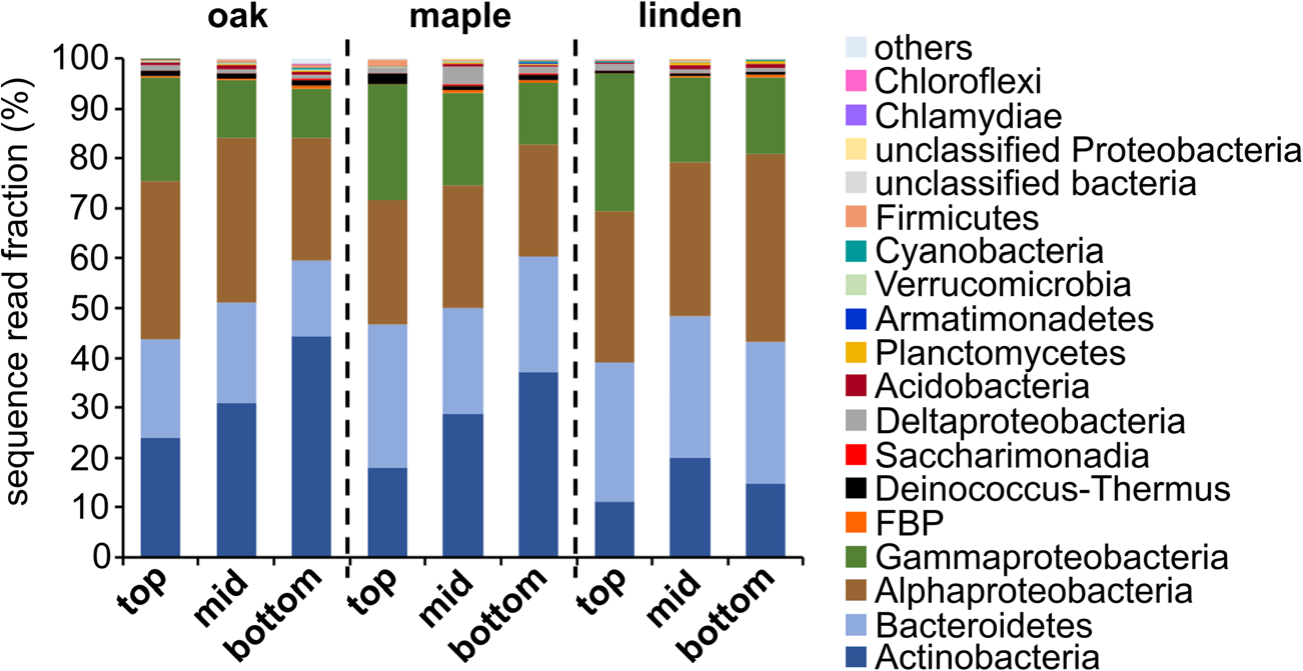
Composition of the bacterial communities associated with the phyllosphere of oak (*Q. robur* L.), maple (*A. pseudoplatanus* L.), and linden (*T. cordata* MILL.) at the top, mid, and bottom positions of the canopy. Each bar represents mean values of three tree individuals and three spatial replicates per tree individual. Taxonomic affiliation is shown on the phylum level or class level for *Proteobacteria* and *Patescibacteria*

The phyllosphere bacterial communities associated with all three tree species were largely dominated by Actinobacteria, Bacteroidetes, Alphaproteobacteria, and Gammaproteobacteria which together accounted for at least 95% of the sequence reads in each sample (Fig. 3). Members of Deinococcus-Thermus, candidate phylum FBP, representatives of the Candidate Phyla Radiation [35] such as *Cand*. Saccharimonadia, and Deltaproteobacteria were consistently present in the phyllosphere communities but rarely reached relative abundances of more than 3%. Taxa associated with chemolithoautotrophic lifestyles within the Gammaproteobacteria, such as *Nitrosomonas*, *Nitrosospira*, or *Ferribacterium*, were only represented by a few sequence reads across all samples.

Notably, we observed major shifts in the relative fractions among the four dominant phyla in dependence of canopy position. For both maple and oak, the fraction of Actinobacteria increased strongly from the top of the canopy (18 and 24%, respectively) to 37 and 44% at the bottom of the canopy (linear regression, *p* < 0.006) while no such height-dependent trend was visible in association with linden trees (*p* = 0.55375) (Fig. 3; Supplementary Fig. 3). Moreover, relative abundances of Actinobacteria were lower in the linden phyllosphere compared to maple and oak for the bottom position of the canopy (pairwise Mann-Whitney *U* test; *p* = 0.008 and *p* = 0.006, respectively) and, compared to oak, also for the top position of the canopy (*p* = 0.004). In turn, the relative fraction of Gammaproteobacteria mostly represented by *Burkholderiacaea*, *Enterobacteriacaea*, *Diplorickettsiacaea*, and *Pseudomonadaceae* tended to decrease from the top towards the bottom of the canopy for all three tree species (Fig. 3); however, these trends were not significant (Supplementary Fig. 3). Bacteroidetes, represented mostly by the families of *Hymenobacteraceae* and *Spirosomacea*e, did not show any obvious changes in relative abundance with canopy position on the phylum level.

Following changes in relative abundances of the most abundant 20 OTUs across the top, mid, and bottom positions of the canopy further demonstrated that the distribution patterns of individual OTUs were often linked to canopy position, which was further modulated by tree species identity. The strongest increase in relative abundance from the top towards the canopy mid and bottom was observed for OTU01 affiliated with *Friedmaniella* (*Propionibacteriaceae*), which constituted a dominant member of the phyllosphere community, accounting for up to 46% of the sequence reads in the individual samples at the canopy bottom and mid position but on average only for 4–11% at the top of the canopy (Supplementary Fig. 4). In turn, several OTUs decreased in relative abundance towards the mid and bottom position of the canopy for all three tree species, e.g., OTU09 (*Massilia*), OTU10 (*Hymenobacter*), OTU15 (*Methylobacterium*), and OTU17 (*Kineococcus*).

**Fig. 4:**
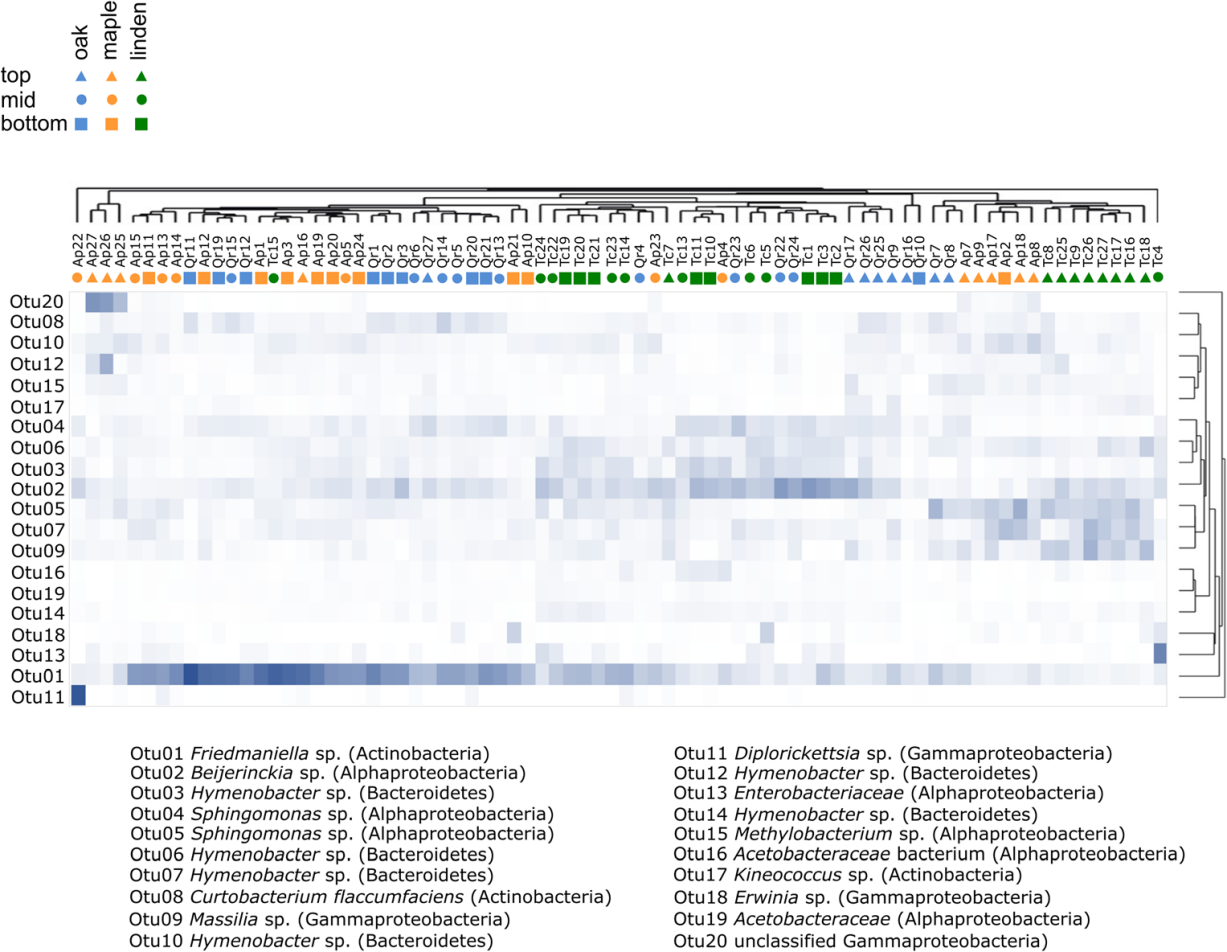
Relative abundance of the 20 most abundant species-level OTUs across all samples. Affiliation with top, mid, or bottom positions of the canopy is depicted by triangles, circles, or squares, respectively. Names of samples refer to microbial communities in association with *A. pseudoplatanus* L. (Ap), *Q. robur* L. (Qr), and *T. cordata* MILL. (Tc). Two-way hierarchical clustering was performed based on Bray-Curtis dissimilarities

Hierarchical clustering based on Bray-Curtis dissimilarities of these 20 OTUs across all samples provided further insight into their preferential association with canopy position or a particular tree species. In general, clustering patterns according to tree species appeared to be less pronounced than those according to canopy position and confirmed the preferential association of OTU01 with bottom and mid canopy positions of oak and maple and its clear distinction from the distribution patterns of the other abundant OTUs (Fig. 4). OTU02, OTU03, OTU04, OTU05, OTU06, OTU07, and OTU09 affiliated with *Beijerinckiaceae*, *Sphingomonadaceae*, *Hymenobacter*, and *Massilia* showed distribution patterns complementary to those of OTU01. OTU05 (*Sphingomonas*), OTU07 (*Hymenobacter*), and OTU09 (*Massilia*) occurred primarily in association with the canopy top position across all three tree species. Clustering of bacterial communities according to tree species was more pronounced for the canopy top position compared to mid and bottom position.

### Phyllosphere Core Microbiome and Co-occurrence Networks

Integrating across tree individuals and positions in the canopy, we further compared the sets of OTUs associated with a given tree species to each other. For each tree species, about 55–57% of the OTUs were unique to that tree species, while a fraction of 26–29% was shared between all three tree species. Maple and oak shared a slightly higher fraction of OTUs between their phyllosphere microbiomes (36–37%) compared to the fraction shared with linden (33–34%) (Supplementary Fig. 5).

Thirty species-level OTUs from four different phyla and 13 different families were present across all tree species and individuals, canopy positions, and spatial replicates, forming the phyllosphere core microbiome. Altogether, these 30 OTUs accounted for 77% of the sequence reads but only for 0.3% of the observed phyllosphere bacterial diversity, indicating that the phyllosphere communities were strongly dominated by these core microbiome representatives. Among the core microbiome members, *Hymenobacteraceae* (*Cytophagales, Bacteroidetes*) contributed the largest number of OTUs, followed by *Burkholderiaceae* (*Betaproteobacteriales*, Gammaproteobacteria) and *Beijerinckiacaea* (*Rhizobiales*, Alphaproteobacteria) (Supplementary Table 4).

**Fig. 5:**
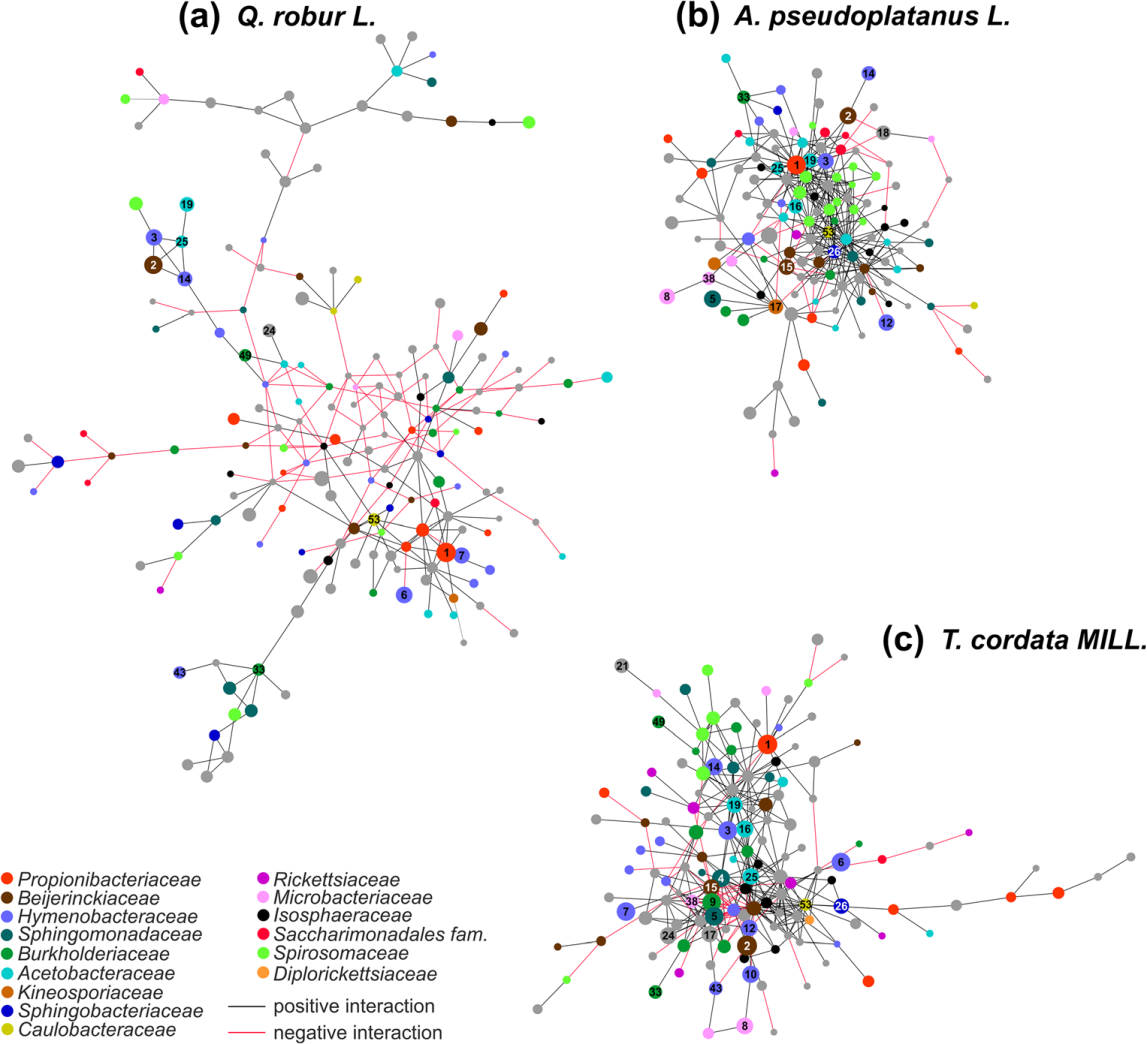
Co-occurrence network of bacterial species-level OTUs across height levels and individuals for each tree species. **a** *Q. robur* L., **b** *A. pseudoplatanus* L., **c** *T. cordata* MILL. Only network modules with more than 10 OTUs are shown. Color of circles denotes taxonomic affiliation on the family level. Numbers indicate core OTUs

Bacterial communities associated with a given tree species were subjected to co-occurrence network analysis, which revealed substantially different networks for each tree species (Fig. 5). We obtained networks with 156 nodes and 332 links for maple, 216 nodes and 258 links for oak, and 161 nodes and 365 links for linden. Notably, co-occurrence networks of the oak phyllosphere microbiomes showed the largest fraction of negative interactions (44.2%), while negative interactions accounted for only 11.1 or 20.3%, respectively, of all interactions in the phyllosphere OTU network of maple and linden. Across samples, OTU01 was not only the OTU with the highest relative abundance but also among the top five OTUs with the highest number of links to other OTUs within the maple and oak canopy. In contrast, OTU01 was less strongly connected in the phyllosphere of linden*,* coinciding with its lower relative abundance in association with that tree species. Overall, OTU01 exhibited mostly positive links to other OTUs. However, these associated OTUs differed substantially across tree species. For the oak phyllosphere, 63% of the OTUs associated with OTU01 were also *Actinobacteria*, while Proteobacteria dominated the associated OTUs in the linden phyllosphere. In association with maple, OTU01 exhibited the most diverse connections to other OTUs, including an especially high contribution of *Spirosomacea* and *Saccharimonadales* compared to the other two tree species.

## Discussion

Phyllosphere microbiota in tree canopies play central roles in biogeochemical cycling and contribute to host plant fitness, protection and productivity [3]. Here, we hypothesized that both position in the canopy and tree species identity shape the phyllosphere microbial community in a floodplain hardwood forest in central Germany. In fact, we found evidence of an influence of both factors; however, canopy position-dependent effects were more pronounced and pointed to vertical gradients across the canopy. Bacterial abundance and OTU richness were lower at the top of the canopy compared to the mid canopy and bottom canopy positions across all three tree species—*Q. robur* L., *A. pseudoplatanus* L., *T. cordata* MILL.. While previous studies reported a large variation of phyllosphere bacterial community structure within a single tree canopy [19, 20], trends of increasing diversity from the top towards the mid and bottom canopies have rarely been described for bacterial [21] or fungal communities [36–38]. Here, we demonstrate that not only microbial diversity but also microbial abundances follow the same trends and are likely affected by the same canopy position-dependent factors.

Leaf-associated bacteria might simply be washed off with rainwater, leading to continuous loss of biomass and OTUs to the mid and bottom positions of the canopy or eventually resulting in their transport to the soil via throughfall or stemflow [39]. In fact, Stone and Jackson [21] found that rainfall influenced compositional similarity of bacterial communities throughout the canopy of *Magnolia* trees, which could also be one the mechanisms underlying the higher similarity between mid canopy and bottom canopy communities versus communities at the top of the canopy observed in our study. Similarly, interior canopy and upper canopy communities were the most distinct in the *Magnolia* canopy [21]. However, these authors also reported that rain did not have any effect on bacterial species richness, questioning to which extent rainfall imposes physical disturbance on the phyllosphere-associated microbiota. Moreover, leaf-associated bacteria are protected against such physical forces by aggregates and biofilms and also by the surface structure of the leaves [40]. Consequently, rainfall is likely not the main driver of lower abundance and diversity at the top of the canopy in our study.

Alternatively, lower diversity at the top of the canopy could be a direct result of harsher environmental conditions acting on the microbial colonizers, such as higher exposure to UV radiation, weather extremes, desiccation, and depletion of nutrients due to rain-mediated wash off [21]. These conditions could lead to a selective enrichment of specialists at the top of the canopy, while the phyllosphere bacteria which are highly abundant at the mid and bottom canopy positions become less competitive under the conditions at the top of the canopy. Interestingly, Actinobacteria, in particular one OTU affiliated with the genus *Friedmaniella*, appeared to be the most responsive bacterial group to canopy position and showed a strong increase in its relative abundance from the top towards the mid and bottom canopy positions. In general, distribution patterns of the 20 most abundant OTUs appeared to be strongly linked to canopy position, suggesting contrasting ecological preferences for bacteria related to *Friedmaniella* versus those related to *Hymenobacter*, *Methylobacterium*, *Kineococcus*, or *Massilia*, whose relative abundance increased at the top of the canopy. Previous findings suggested that general stress response is an essential mechanism for plant colonization by *Methylobacterium*, including responses to heat shock and desiccation, and oxidative, UV, ethanol, and osmotic stresses [11, 41]. In addition, increased abundances of *Methylobacterium* in upper parts of the canopy of *Magnolia* trees have been explained by a positive response of this genus to changes in leaf physiology following higher light or higher temperature, or by a direct response to these environmental parameters [21]. In addition to a better adaption to harsh environmental conditions at the top of the canopy, increased relative abundances of *Methylobacterium*, *Hymenobacter*, *Kineococcus*, and *Massilia* may also have been supported by reduced abundances of *Friedmaniella* as a potentially very competitive inhabitant of the hardwood forest phyllosphere.

Plant species identity was identified as another key factor that influenced phyllosphere bacterial community composition in the floodplain forest, similar to tropical forests and temperate forest ecosystems in North America [42–44]. Bacterial communities associated with oak and maple were more similar to each other than to those associated with linden trees, suggesting that oak and maple provided more favorable and more similar conditions for certain taxa, e.g., for Actinobacteria related to *Friedmaniella,* than did linden. Plant host attributes such as plant taxonomic identity and phylogeny, wood density, leaf mass per area, seed mass, leaf water content, and leaf nitrogen and phosphorus concentrations have been suggested as key factors underlying the relationship between plant species and their microbiome in neotropical as well as in temperate forests [43, 45, 46]. Additional factors may include the rate of production of volatile organic compounds such as methanol, which can act as important substrate for the phyllosphere microbiota [7, 42, 47, 48].

Co-occurrence network analysis revealed that the three tree species did not only differ in their bacterial community structure and OTU composition but also in the patterns how these OTUs were connected to each other across tree individuals, canopy position, and spatial replicates. The observed larger fraction of negative interactions between OTUs in association with oak may point to stronger vertical gradients within the oak canopy or larger variation across individuals of the same tree species. Moreover, OTUs with a central position in the network, e.g., by multiple connections to other OTUs, differed between tree species. These findings suggest that plant host-related factors and the chemical environment that they shape select for specific microbial core consortia that are strongly tree species-dependent.

Since Actinobacteria made up to 65% of the phyllosphere bacterial community in some cases, they were by far more prominent in our study compared to neotropical or tropical forests [43, 48] but also to previous investigations of Canadian temperate forests or the phyllosphere of hornbeam (*Carpinus betulus*), where they only accounted for 5–9% of the total community [46, 49]. The most abundant OTU in our study was closely related to *Friedmaniella okinawensis* and *F. sagamiharensis* originally isolated from spider webs in a Japanese forest [50]. Bacteria related to *Friedmaniella* have been found in lower abundances in the phyllosphere of apple orchards or in urban environments [51, 52] and can also grow as endophytes [53, 54]. In fact, species within the genus *Friedmaniella* isolated from forest spider webs or the bark of mangrove plants have the capability to utilize a broader range of organic carbon compounds than other species of that genus [50, 53], suggesting that this broader substrate spectrum could be one the mechanisms underlying their success in the phyllosphere. Alternatively, the high relative abundance of *Friedmaniella* could also be linked to a stage of early senescence of the leaves, since Actinobacteria are generally known as major decomposers of leaf litter [55].

Most of the other genus-level taxa representing the hardwood forest core microbiome, such as *Hymenobacter*, *Methylobacterium*, *Sphingomonas*, and *Pseudomonas* have frequently been observed in association with other temperate forest tree species [19, 21] but also with herbaceous plants [16]. The genus *Methylobacterium* uses methanol as its carbon and energy source, a C1 compound typically released by plants [56]. Besides methanol, small amounts of nutrients, such as glucose, fructose, and sucrose [1], but also amino acids, methane, terpenes, and chloromethane [16, 49, 57, 58] can leach from the interior of the plant and be available for the phyllosphere microbiota. Overall, our findings suggest that microbial processes in the hardwood forest canopies are largely dominated by heterotrophic or C1-dependent metabolisms. Although nitrification has previously been proposed as an important process in tree canopies, stimulated by excess atmospheric deposition of ammonia [6, 59], we found only few sequence reads affiliated with chemolithoautotrophic *Nitrosomonadaceae*.

Given the temporal development of forest tree canopies throughout the growing season, our sampling provides only one snapshot, and the extent to which the September phyllosphere communities differ from earlier stages in spring and summer remains currently unclear. A previous study in a temperate mixed forest showed that temporal effects were smaller than those associated with host species identity [46]. Successional changes in phyllosphere communities can be associated with changes in the physiology of the host plant but can also be shaped by the constant import of microbes from various sources such as air, soils, rainwater, and animal and plant dispersal vectors [43, 60, 61]. Representatives of the genera *Hymenobacter*, *Methylobacterium*, and *Massilia* have been reported from air samples [62] or from aerosols originating from agricultural practices [7, 63], suggesting that airborne microbes play a major role in the early colonization of the surfaces of young leaves in spring [7] and could continuously be introduced to the phyllosphere communities throughout the season. Consequently, the September phyllosphere represents a stage that integrates the results of different mechanisms of colonization and competitive interactions between phyllosphere microbiota throughout the growing season.

## Conclusions

Our findings clearly demonstrate that both position in the canopy and tree species have a strong effect on the structure of phyllosphere bacterial communities in a floodplain hardwood forest. Consistently lower bacterial diversity at the top of the canopy compared to the canopy mid and bottom positions pointed to a stronger selective pressure on phyllosphere bacteria given presumably harsher environmental conditions at the treetop. Across all three tree species, we observed a striking predominance of Actinobacteria related to *Friedmaniella* sp., which could be a typical feature of floodplain hardwood forests or linked to the early senescent state of leaves sampled in mid September.

## Electronic supplementary material

ESM 1 (PDF 813 kb)
