# Supplementary material for: Canopy Position Has a Stronger Effect than Tree Species Identity on Phyllosphere Bacterial Diversity in a Floodplain Hardwood Forest
Source: Microb Ecol. 2020 Aug 6;81(1):157–68. doi: 10.1007/s00248-020-01565-y (PMC7794210; doi:10.1007/s00248-020-01565-y)
Supplement: Supplementary file 1 — (PDF 813 kb) [file 248_2020_1565_MOESM1_ESM.pdf]

Supplementary Material

Canopy position has a stronger effect than tree species identity on phyllosphere bacterial diversity in a floodplain hardwood forest

Martina Herrmann[1,2*], Patricia Geesink[1], Ronny Richter[2, 3,4], Kirsten Küsel[1,2]

[1]Institute of Biodiversity, Aquatic Geomicrobiology, Friedrich Schiller University Jena, Dornburger Strasse 159, D-07743 Jena, Germany

[2]German Center for Integrative Biodiversity Research, Deutscher Platz 5e, 04103 Leipzig, Germany

[3]Systematic Botany and Functional Biodiversity, Institute for Biology, Leipzig University, Johannisallee 21, 04103 Leipzig

[4]Geoinformatics and Remote Sensing, Institute of Geography, Johannisallee 19a, Leipzig University, 04103 Leipzig, Germany

*Corresponding author:

Dr. Martina Herrmann

Friedrich Schiller University Jena

Institute of Biodiversity – Aquatic Geomicrobiology

Dornburger Strasse 159

D-07743 Jena

Phone: +49 (0)3641 949459

Email: martina.herrmann@uni-jena.de

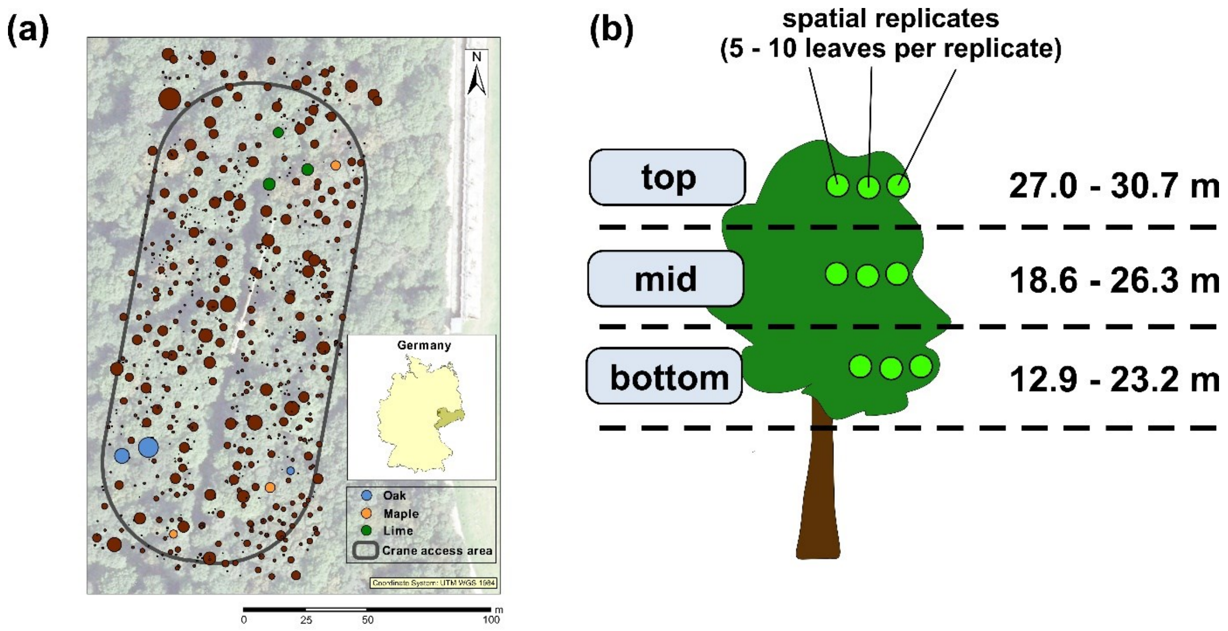

Supplementary Fig. 1:

Study site and sampling design. (a) Location of the tree individuals sampled in this study within the total canopy crane research site. Tree species are distinguished by color. (b) Sampling design. Samples were taken in triplicates from the top, mid and bottom position of the canopy.

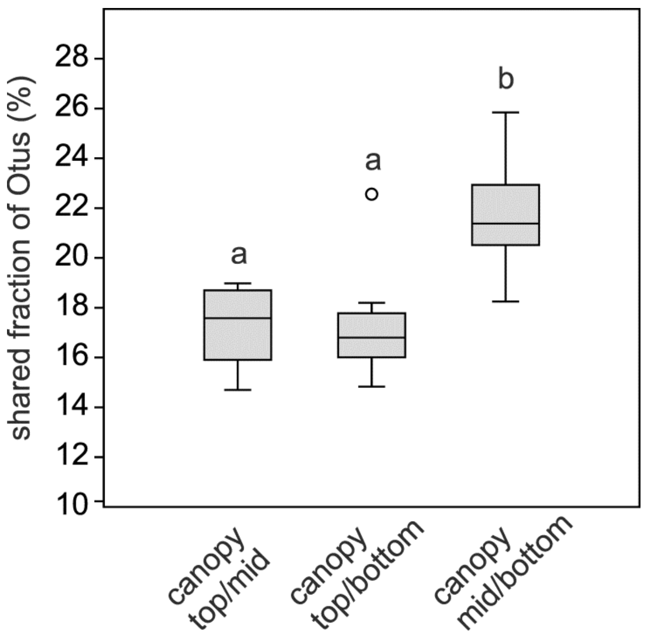

Supplementary Fig. 2:

Fraction of OTUs shared between the canopy's top and mid position, top and bottom position, and mid and bottom position, respectively across all three tree species. Box plots represent mean values calculated from three spatial replicates per tree individual and position within the canopy.

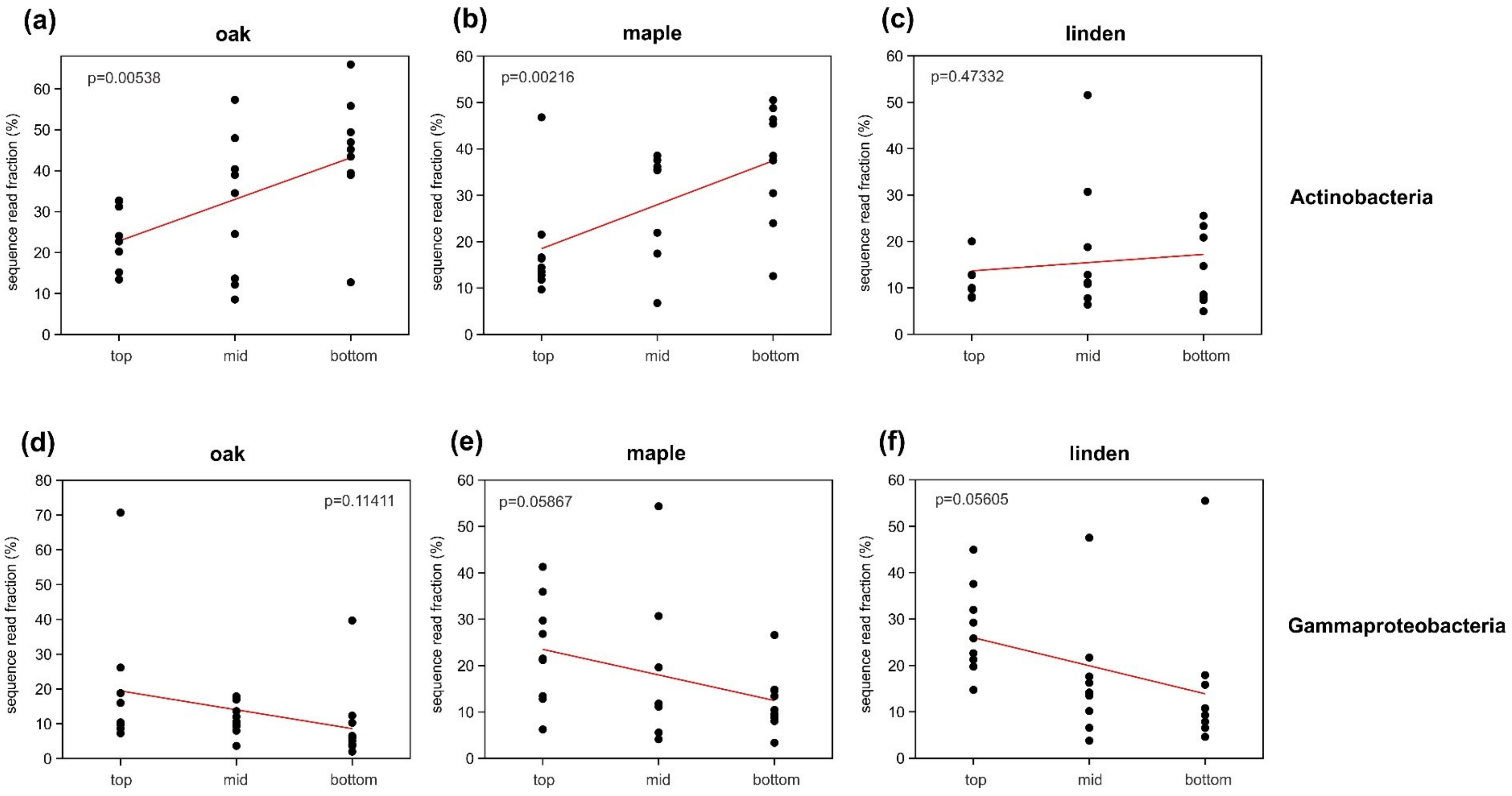

Supplementary Fig. 3

Linear regression of changes in relative abundances of Actinobacteria (a-c) and Gammaproteobacteria (d-f) from the top position of the canopy via mid position to the bottom of the canopy. Analyses were performed separately for each tree species. Data points originate from three individuals of a given tree species with three spatial replicates per position in the canopy.

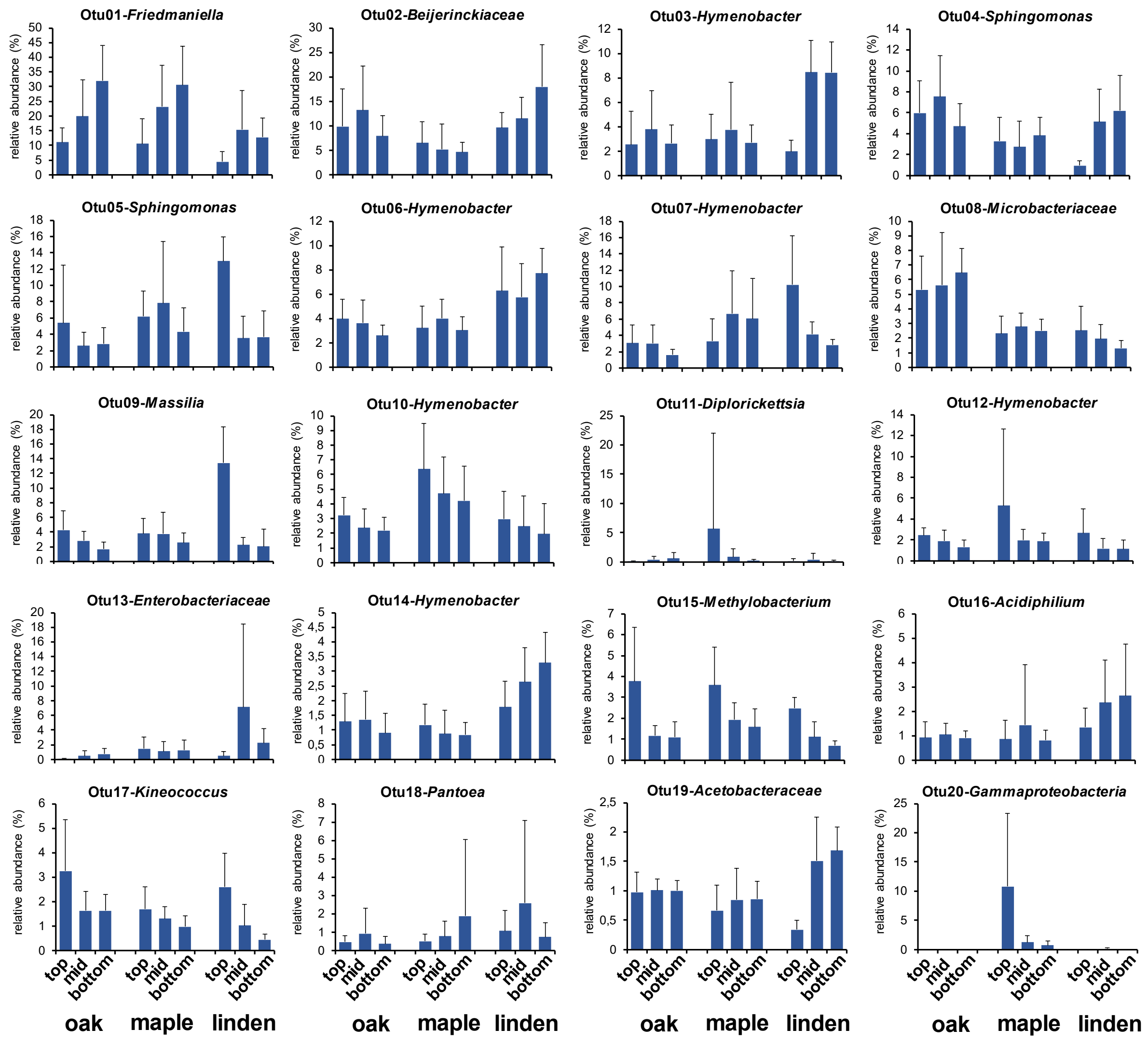

Supplementary Fig. 4:

Changes in relative abundances of the first 20 most abundant OTUs across top, mid, and bottom canopy positions, shown separately for the three tree species. Data are mean (± standard deviation) of three individuals of a tree species, sampled in three spatial replicates per canopy position.

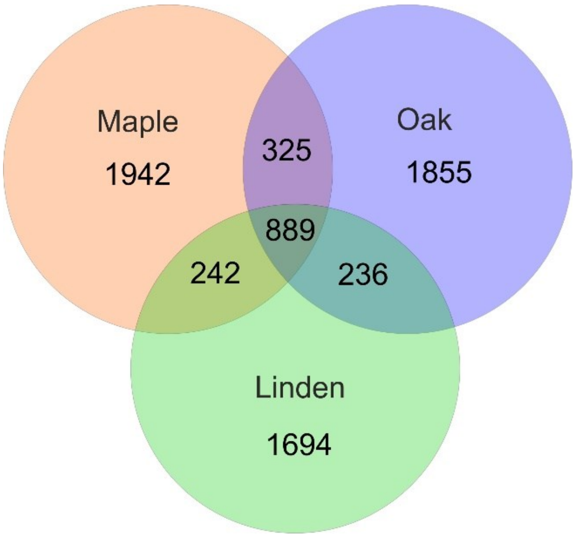

Supplementary Fig. 5:

Number of species-level OTUs shared between tree species. OTUs associated with maple, oak, and linden across individuals, canopy positions, and spatial replicates were merged to one OTU pool per tree species, and comparisons of shared OTUs were made between these tree-species specific OTU pools.

Supplementary Table 1:

Estimated age of the tree individuals sampled in this study based on stem diameter measured on breast level (BHD). For all species, age was estimated based on locally calibrated growth models. Since accurate tree ring dating was not possible for *T. cordata*, age was predicted based on average tree ring width.

Tree ID	species	BHD (cm)	predicted age (y)	minimum age (y)
K129.0	*Quercus robur L.*	133.7	370	-
K317.0	*Quercus robur L.*	86.5	195	-
K733.0	*Quercus robur L.*	53.6	102	-
K33.0	*Acer pseudoplatanus L.*	55.4	97	-
K513.1	*Acer pseudoplatanus L.*	64.7	127	-
K754.0	*Acer pseudoplatanus L.*	70	146	-
K444.0	*Tilia cordata MILL.*	81.5	240	151
K455.0	*Tilia cordata MILL.*	69.6	205	129
K517.1	*Tilia cordata MILL.*	79.9	235	148

Supplementary Table 2:

Abundances of bacterial 16S rRNA genes per g (dry weight) in association with leaves of oak, maple, and linden at top, mid, and bottom canopy positions. Data are means and standard deviation (in parentheses) of three technical qPCR replicates. For a number of samples, leaf dry weight is not available due to loss of plant material after DNA extraction and thus, gene abundances could not be determined (n. a.).

**tree species**	**canopy position**	**sample**								
		**Qr7**	**Qr8**	**Qr9**	**Qr16**	**Qr17**	**Qr18**	**Qr25**	**Qr26**	**Qr27**
oak	top	n. a.	n. a.	9.3 x 10^{6} (7.0 x 10^{5})	n. a.	n. a.	1.9 x 10^{6} (1.5 x 10^{5})	n. a.	n. a.	1.9 x 10^{7} (9.6 x 10^{5})
		**Qr4**	**Qr5**	**Qr6**	**Qr13**	**Qr14**	**Qr15**	**Qr22**	**Qr23**	**Qr24**
oak	mid	n. a.	2.7 x 10^{7} (6.6 x 10^{5})	3.2 x 10^{7} (1.5 x 10^{6})	1.5 x 10^{8} (1.9 x 10^{7})	1.3 x 10^{8} (6.1 x 10^{6})	6.1 x 10^{7} (6.4 x 10^{6})	8.0 x 10^{7} (3.6 x 10^{6})	1.3 x 10^{8} (6.6 x 10^{6})	6.7 x 10^{8} (1.9 x 10^{7})
		**Qr1**	**Qr2**	**Qr3**	**Qr10**	**Qr11**	**Qr12**	**Qr19**	**Qr20**	**Qr21**
oak	bottom	8.1 x 10^{7} (8.3 x 10^{6})	2.3 x 10^{8} (1.5 x 10^{7})	9.5 x 10^{7} (3.8 x 10^{6})	7.8 x 10^{7} (4.1 x 10^{6})	1.6 x 10^{8} (3.9 x 10^{6})	1.6 x 10^{8} (4.3 x 10^{6})	n. a.	n. a.	n. a.
		**Ap7**	**Ap8**	**Ap9**	**Ap16**	**Ap17**	**Ap18**	**Ap25**	**Ap26**	**Ap27**
maple	top	n. a.	n. a.	n. a.	n. a.	n. a.	7.9 x 10^{7} (2.2 x 10^{6})	n. a.	n. a.	2.2 x 10^{7} (1.0 x 10^{6})
		**Ap4**	**Ap5**	**Ap6**	**Ap13**	**Ap14**	**Ap15**	**Ap22**	**Ap23**	**Ap24**
maple	mid	4.0 x 10^{8} (3.4 x 10^{7})	6.8 x 10^{7} (5.5 x 10^{6})	4.5 x 10^{7} (5.0 x 10^{6})	9.1 x 10^{7} (7.1 x 10^{6})	2.4 x 10^{8} (1.3 x 10^{7})	2.0 x 10^{8} (1.7 x 10^{6})	3.0 x 10^{8} (7.4 x 10^{6})	9.5 x 10^{7} (1.0 x 10^{7})	1.3 x 10^{8} (7.4 x 10^{6})
		**Ap1**	**Ap2**	**Ap3**	**Ap10**	**Ap11**	**Ap12**	**Ap19**	**Ap20**	**Ap21**
maple	bottom	2.4 x 10^{8} (7.3 x 10^{6})	n. a.	n. a.	2.6 x 10^{8} (1.1 x 10^{7})	n. a.	n. a.	2.5 x 10^{8} (6.2 x 10^{6})	n. a.	2.1 x 10^{8} (8.8 x 10^{6})

Supplementary Table 2, continued

tree species	canopy position	sample								
		**Tc7**	**Tc8**	**Tc9**	**Tc16**	**Tc17**	**Tc18**	**Tc25**	**Tc26**	**Tc27**
linden	top	n. a.	n. a.	1.2×10^{8} (6.9×10^{6})	n. a.	n. a.	5.0×10^{7} (4.8×10^{6})	n. a.	n. a.	7.4×10^{7} (3.5×10^{6})
		**Tc4**	**Tc5**	**Tc6**	**Tc13**	**Tc14**	**Tc15**	**Tc22**	**Tc23**	**Tc24**
linden	mid	3.1×10^{8} (2.6×10^{7})	3.7×10^{8} (4.2×10^{6})	2.1×10^{8} (1.6×10^{7})	n. a.	2.8×10^{8} (1.5×10^{7})	2.4×10^{8} (2.4×10^{7})	2.1×10^{8} (1.9×10^{7})	2.1×10^{8} (2.0×10^{7})	n. a.
		**Tc1**	**Tc2**	**Tc3**	**Tc10**	**Tc11**	**Tc12**	**Tc19**	**Tc20**	**Tc21**
linden	bottom	3.7×10^{8} (1.4×10^{7})	2.4×10^{8} (1.2×10^{7})	1.6×10^{8} (1.2×10^{7})	5.1×10^{8} (2.8×10^{7})	7.6×10^{8} (8.2×10^{7})	n. a.	1.3×10^{9} (7.7×10^{7})	7.9×10^{8} (7.3×10^{7})	5.3×10^{8} (3.4×10^{7})

Supplementary Table 3:

Results of PERMANOVA analysis based on Bray-Curtis dissimilarities testing the effect of canopy position and tree species identity on phyllosphere bacterial community composition. Pr(>F), p value.

variable	F value	R^2	Pr(>F)
canopy position	8.5038	0.15218	0.001***
tree species	8.3156	0.14881	0.001***
canopy position:tree species	2.2809	0.08183	0.001***

Supplementary Table 4:

Taxonomic affiliation of the 30 OTUs forming the phyllosphere core microbiome.

OTU	Phylum (according to SILVA reference taxonomy v132)	Family (according to SILVA reference taxonomy v132)	Closest BLAST hit (sequence identity/%)	Fraction of sequence reads across all samples (%)
Otu01	Actinobacteria	*Propionibacteriaceae*	MH813394.1 *Friedmaniella* sp. strain CP13 (100)	17.7
Otu02	Proteobacteria	*Beijerinckiacaea*	MN416236.1 *Beijerinckia* sp. strain KIGAM230 (100)	9.6
Otu03	Bacteroidetes	*Hymenobacteraceae*	KU097332.1 *Hymenobacter* sp. 14-57-B6 (98.6)	4.2
Otu04	Proteobacteria	*Sphingomonadaceae*	MT386184.1 *Sphingomonas* sp. strain Atecer8B (100)	4.5
Otu05	Proteobaceria	*Sphingomonadaceae*	MT585898.1 *Sphingomonas* sp. strain 2176 (100)	5.4
Otu06	Bacteroidetes	*Hymenobacteraceae*	JF742943.1 *Cytophagaceae* bacterium LCK-2009.32 (99.1)	4.4
Otu07	Bacteroidetes	*Hymenobacteraceae*	KC763792.1 *Hymenobacter* sp. MIC2056 (98.3)	4.5
Otu08	Actinobacteria	*Microbacteriaceae*	CP041259.1 *Curtobacterium flaccumfaciens* pv. *flaccumfaciens* strain Cff1037 (100)	3.4
Otu09	Proteobacteria	*Burkholderiaceae*	MT367291.1 *Massilia* sp. strain KACC21261 (100)	4.1
Otu10	Bacteroidetes	*Hymenobacteraceae*	KU097332.1 *Hymenobacter* sp. 14-57-B6 (98.6)	3.4
Otu12	Bacteroidetes	*Hymenobacteraceae*	MN784633.1 *Hymenobacter* sp. RP-2-7 (97.6)	2.2
Otu14	Bacteroidetes	*Hymenobacteraceae*	MH549147.2 *Hymenobacter* sp. strain Fur1 (96.7)	1.6
Otu15	Proteobacteria	*Beijerinckiaceae*	MT360236.1 *Methylobacterium* sp. strain YMA23 (100)	1.9
Otu16	Proteobacteria	*Acetobacteraceae*	JX458433.1 *Acetobacteraceae* bacterium W1.09-37 (98.8)	1.4
Otu17	Actinobacteria	*Kineosporiaceae*	MN989067.1 *Kineococcus* sp. strain B3K081a (100)	1.6
Otu18	Proteobacteria	*Enterobacteriaceae*	LC333546.1 *Erwinia* sp. fn 89 (100)	1.0
Otu19	Proteobacteria	*Acetobacteraceae*	KX990258.1 *Acetobacteraceae* bacterium strain Ap43E (98)	1.0
Otu21	Deinococcus-Thermus	*Deinococcaceae*	MK752552.1 *Deinococcus* sp. strain BRD123 (98.8)	0.9

Supplementary Table 4, continued

OTU	Phylum (according to SILVA reference taxonomy v132)	Family (according to SILVA reference taxonomy v132)	Closest BLAST hit (sequence identity/%)	Fraction of sequence reads across all samples (%)
Otu22	Proteobacteria	*Burkholderiaceae*	DQ490310.1 *Oxalobacteraceae* bacterium KVD-1921-01 (99.1)	0.9
Otu24	Proteobacteria	*Pseudomonadaceae*	MT585899.1 *Pseudomonas* sp. strain 2180 (100)	0.9
Otu25	Proteobacteria	*Acetobacteraceae*	KY908234.1 *Acidisphaera* sp. strain Br1-6 (96.3)	0.7
Otu26	Bacteroidetes	*Sphingobacteriaceae*	KP099945.1 *Pedobacter luteus* strain HME8461 (95.7)	0.6
Otu33	Proteobacteria	*Burkholderiaceae*	MN989178.1 *Variovorax* sp. strain C4P084 (100)	0.3
Otu34	Actinobacteria	*Kineosporiaceae*	MN493040.1 *Kineosporiaceae* bacterium B12 (100)	0.4
Otu38	Actinobacteira	*Microbacteriaceae*	MT360246.1 *Frondihabitans* sp. strain YMA-33 (100)	0.4
Otu39	Proteobacteria	*Beijerinckiaceae*	MT556429.1 *Methylobacterium* sp. strain B20A6 (100)	0.3
Otu43	Bacteroidetes	*Hymenobacteraceae*	KC763792.1 *Hymenobacter* sp. MIC2056 (98.8)	0.3
Otu49	Proteobacteria	*Burkholderiaceae*	KR922299.1 *Burkholderiales* bacterium PDD-69b-30 (99.3)	0.2
Otu53	Proteobacteria	*Caulobacteraceae*	KY908306.1 *Caulobacter* sp. strain Os2-9 (98.0)	0.2
Otu70	Actinobacteria	*Microbacteriaceae*	MH127824.1 *Microbacterium hatanonis* strain EB381 (100)	0.1
